# Supplementary material for: The endosomal escape vehicle platform enhances delivery of oligonucleotides in preclinical models of neuromuscular disorders
Source: Mol Ther Nucleic Acids. 2023 Jun 29;33:273–85. doi: 10.1016/j.omtn.2023.06.022 (PMC10393622; doi:10.1016/j.omtn.2023.06.022)
Supplement: Document S1. Figures S1–S3 [file mmc1.pdf]

OMTN, Volume *33*

Supplemental information

The endosomal escape vehicle platform enhances delivery of oligonucleotides in preclinical models of neuromuscular disorders

Xiang Li, Mahboubeh Kheirabadi, Patrick G. Dougherty, Kimberli J. Kamer, Xiulong Shen, Nelsa L. Estrella, Suresh Peddigari, Anushree Pathak, Sara L. Blake, Emmanuelle Sizensky, Carmen del Genio, Arti B. Gaur, Mohanraj Dhanabal, Mahasweta Girgenrath, Natarajan Sethuraman, and Ziqing Qian

A

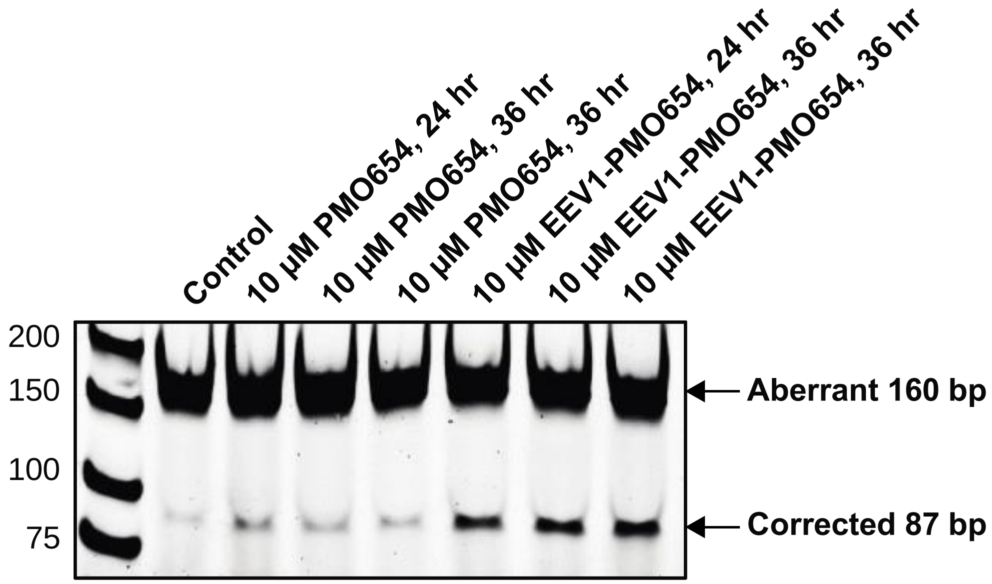

B

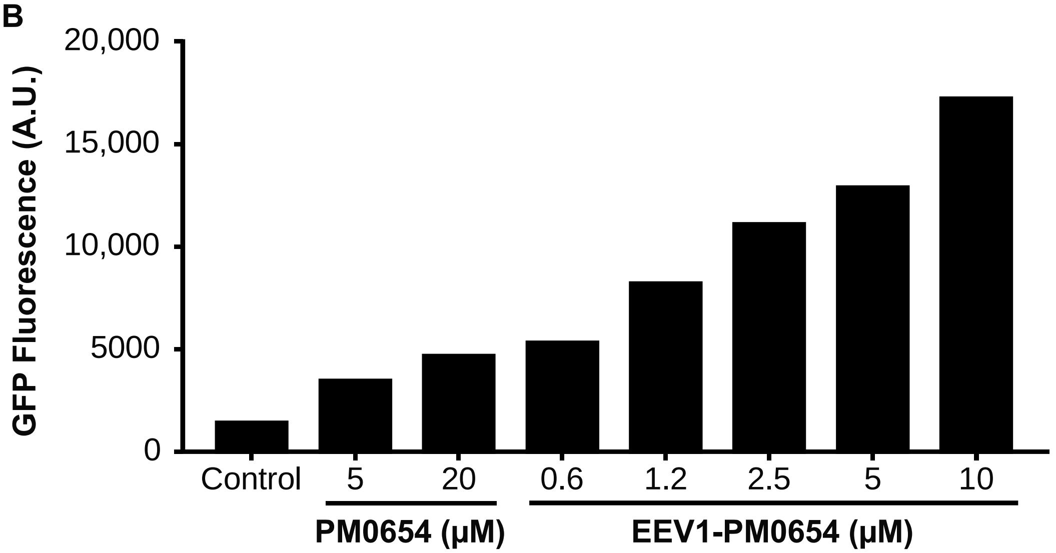

Figure S1. Evaluation of splice-switching activity for the EGFP pre-mRNA in HeLa EGFP-654 cells. (A) RT-PCR analysis of both corrected (87 bp) and uncorrected (160 bp) bands. (B) Restoration of EGFP fluorescence in HeLa EGFP-654 after 24-hour treatment and quantified by flow cytometry with 10,000 events per sample. Data were collected from a single experiment without replicates.

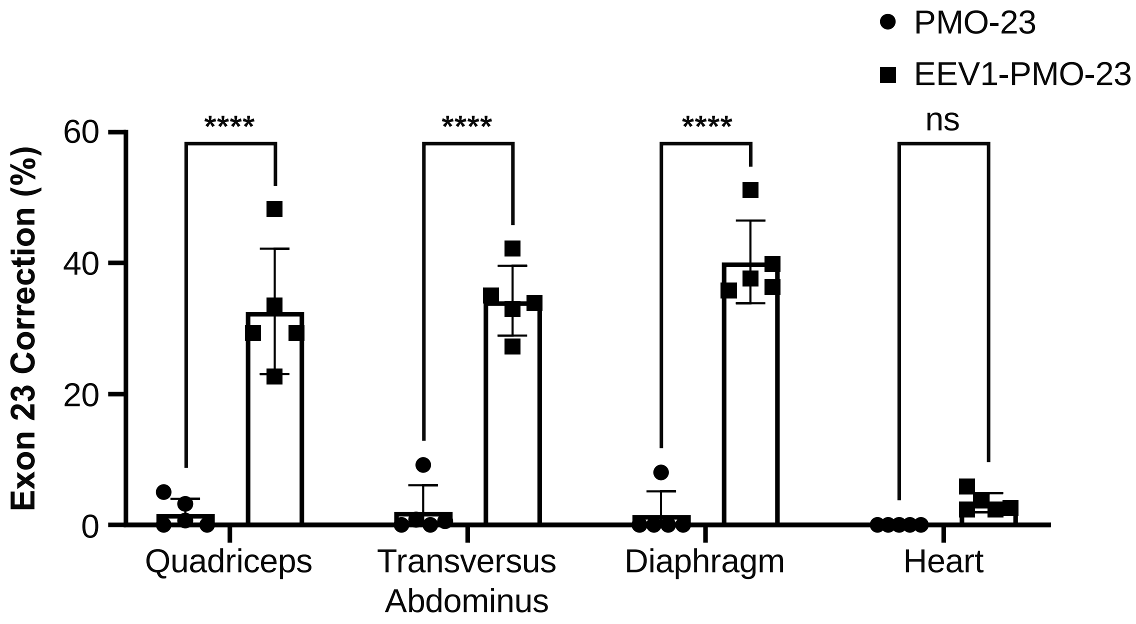

Figure S2. Exon 23–skipping induced by 30 mg/kg PMO or EEV1-PMO-23 (PMO equivalent) in C57BL/10 seven (7) days post-IV injection as quantified by RT-PCR

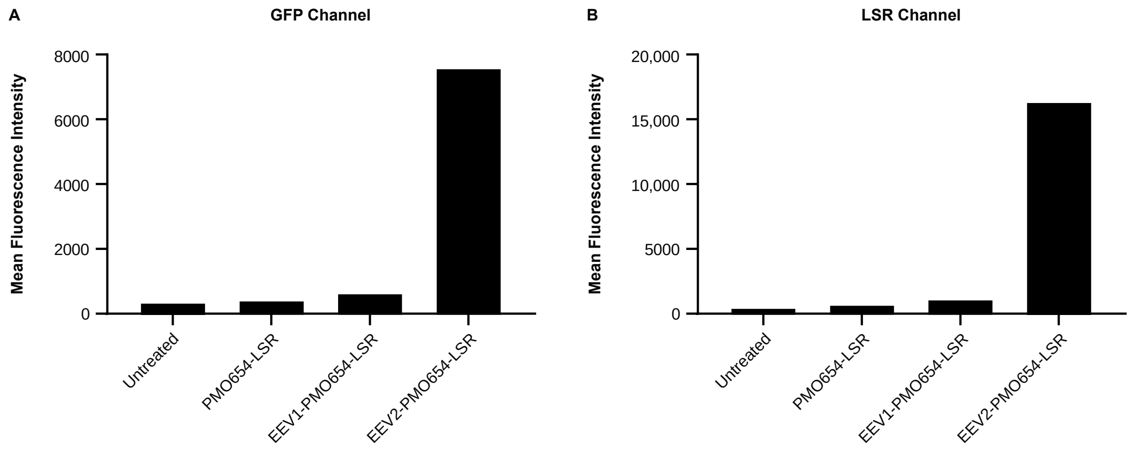

Figure S3. Flow cytometry analysis of both EGFP (A) and Lissamine Rhodamine (B) signals for HeLa-EGFP-654 cells treated with 2 µM Lissamine Rhodamine B (LSR) modified PMO654, EEV1-PMO654 and EEV2-PMO654 with 2000 events per sample. Data were collected from a single experiment without replicates
