# Supplementary material for: The endosomal escape vehicle platform enhances delivery of oligonucleotides in preclinical models of neuromuscular disorders
Source: Mol Ther Nucleic Acids. 2023 Jun 29;33:273–85. doi: 10.1016/j.omtn.2023.06.022 (PMC10393622; doi:10.1016/j.omtn.2023.06.022)
Supplement: Document S2. Article plus supplemental information [file mmc2.pdf]

Molecular Therapy
Nucleic Acids
Original Article

The endosomal escape vehicle platform enhances delivery of oligonucleotides in preclinical models of neuromuscular disorders

Xiang Li,[1,3] Mahboubeh Kheirabadi,[1,3] Patrick G. Dougherty,[1,3] Kimberli J. Kamer,[1] Xiulong Shen,[1] Nelsa L. Estrella,[1] Suresh Peddigari,[1] Anushree Pathak,[1] Sara L. Blake,[1] Emmanuelle Sizensky,[1] Carmen del Genio,[2] Arti B. Gaur,[2] Mohanraj Dhanabal,[1] Mahasweta Girgenrath,[1] Natarajan Sethuraman,[1] and Ziqing Qian[1]

[1]Entrada Therapeutics, One Design Center Place, Suite 17-500, Boston, MA 02210, USA; [2]Dartmouth Hitchcock Medical Center, 1 Medical Center Drive, Lebanon, NH 03756, USA

Biological therapeutic agents are highly targeted and potent but limited in their ability to reach intracellular targets. These limitations often necessitate high therapeutic doses and can be associated with less-than-optimal therapeutic activity. One promising solution for therapeutic agent delivery is use of cell-penetrating peptides. Canonical cell-penetrating peptides, however, are limited by low efficiencies of cellular uptake and endosomal escape, minimal proteolytic stability, and toxicity. To overcome these limitations, we designed a family of proprietary cyclic cell-penetrating peptides that form the core of our endosomal escape vehicle technology capable of delivering therapeutic agent-conjugated cargo intracellularly. We demonstrated the therapeutic potential of this endosomal escape vehicle platform in preclinical models of muscular dystrophy with distinct disease etiology. An endosomal escape vehicle-conjugated, splice-modulating oligonucleotide restored dystrophin protein expression in striated muscles in the *mdx* mouse, a model for Duchenne muscular dystrophy. Furthermore, another endosomal escape vehicle-conjugated, sterically blocking oligonucleotide led to knockdown of aberrant transcript expression levels in facioscapulohumeral muscular dystrophy patient-derived skeletal muscle cells. These findings suggest a significant therapeutic potential of our endosomal escape vehicle conjugated oligonucleotides for targeted upregulation and downregulation of gene expression in neuromuscular diseases, with possible broader application of this platform for delivery of intracellular biological agents.

INTRODUCTION

Antisense oligonucleotides (ASOs) are short, synthetic, single-stranded, non-coding oligonucleotides that can selectively bind target RNAs to regulate splicing and expression. The therapeutic applications of ASOs are extremely broad because these compounds can be synthesized with a specific nucleotide sequence directed against target RNAs. To date, the potential of ASOs as a therapeutic modality has been demonstrated using 9 US Food and Drug Administration (FDA)-approved oligonucleotide drugs across a range of disease indications.[1,2] In Duchenne muscular dystrophy (DMD), approved ASO therapies utilize a stabilized oligonucleotide chemistry known as phosphorodiamidate morpholino oligomers (PMOs) to enable frameshift correction by exon skipping, ultimately resulting in production of a truncated but functional dystrophin protein.[3] The advantages of PMOs over other ASO chemistries include low toxicity, high binding affinity, resistance to degradation, and water solubility.[4] In addition to exon skipping, PMOs can be used to sterically block target sequences, thereby reducing expression. To that end, PMO-mediated approaches are being investigated in facioscapulohumeral muscular dystrophy (FSHD) by targeting the polyadenylation signal region of the double homeobox 4 (*DUX4*) transcript, destabilizing the transcript and reducing its aberrant expression.[5,6]

There are 2 major barriers to attaining high efficacy with ASO therapeutic agents. First, the ASOs must reach and be internalized by the target cell. Second, ASOs must escape endosomal/lysosomal compartments to reach the cytosol or nucleus after cellular entry. In the case of DMD, currently approved ASO-based exon-skipping therapies have modest clinical benefits, especially in cardiac tissues, likely because of limited cellular uptake and poor biodistribution.[7] One approach to enhance intracellular delivery of oligonucleotides is use of cell-penetrating peptides (CPPs).[8] Most CPPs deliver their cargo into the cell via endocytosis and are initially localized in the endosome. The CPP-cargo conjugates must escape from endosomal compartments to reach their intracellular targets. This process, known as endosomal escape, poses a significant barrier to delivery of intracellular therapeutic agents.[9,10] Canonical linear CPPs are generally limited by low efficiencies of cellular uptake and endosomal escape, minimal proteolytic stability, and toxicity. To overcome these shortcomings, we leveraged a family of proprietary cyclic CPPs that form the core

Received 20 January 2023; accepted 26 June 2023;
https://doi.org/10.1016/j.omtn.2023.06.022.

[3]These authors contributed equally

Correspondence: Ziqing Qian, Entrada Therapeutics, 6 Tide Street, Boston, MA 02210, USA.
E-mail: qian@entradatx.com

This is an open access article under the CC BY license (http://creativecommons.org/licenses/by/4.0/).

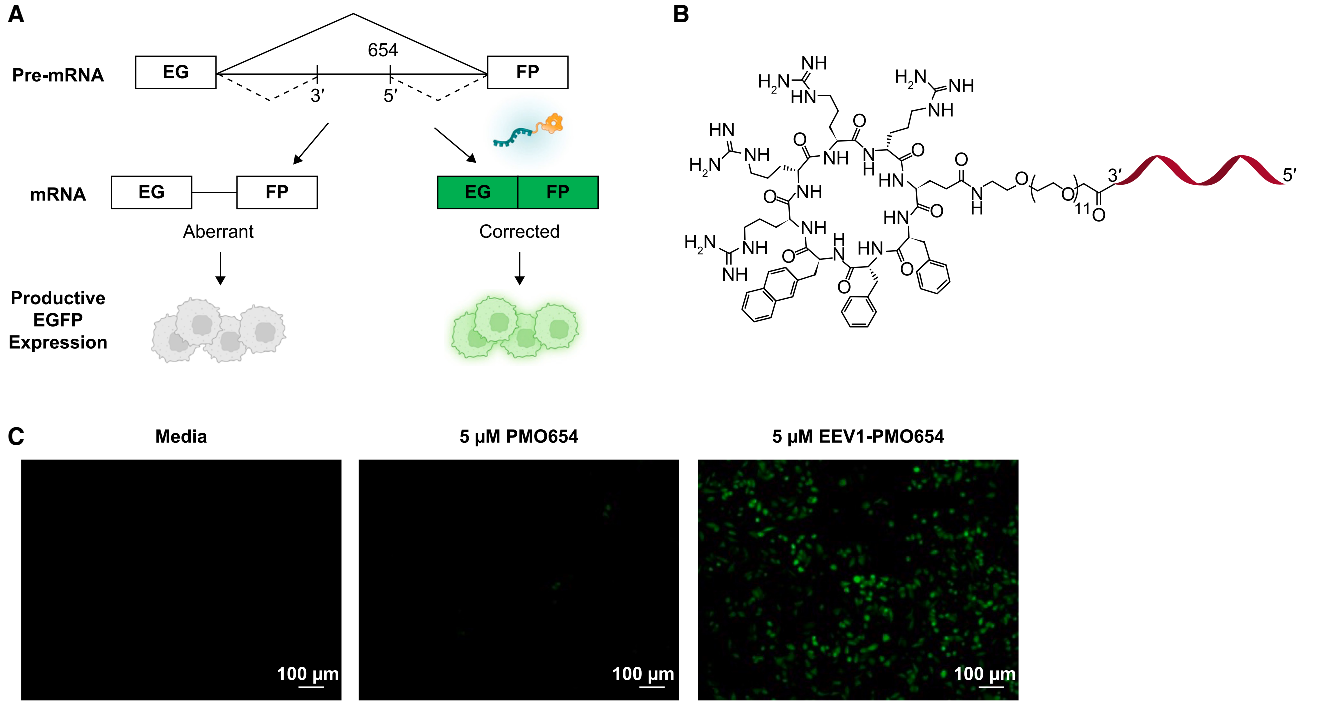

Figure 1. Correction of aberrant splicing in HeLa EGFP-654 cells and by EEV1-PMO654

(A) Schematic overview of the EGFP-654 gene. (B) Structure of EEV1-PMO654 conjugates via amide bond formation. (C) Restoration of EGFP fluorescence in the presence EEV1-PMO654 by live-cell fluorescence microscopy.

of our endosomal escape vehicle (EEV) technology. These cyclic CPPs, first described by Qian et al.,[11] have shown improved proteolytic stability, enhanced cellular uptake, and endosomal escape[12,13] when conjugated to peptides, proteins, and other large molecules.[14,15] The study presented here examined, for the first time, the therapeutic potential of EEV-oligonucleotide conjugates. Our research demonstrates the efficacy and improved delivery of EEV platform-based ASO conjugates in a HeLa EGFP-654 cell line and EGFP-654 transgenic mice. We also illustrate the utility of the EEV platform-based ASO conjugate approach to neuromuscular disorders with 2 examples: (1) modulation of pre-mRNA splicing to promote high levels of exon skipping and dystrophin protein restoration in a DMD mouse model and (2) biomarker transcript reduction in 2 FSHD cell models.

RESULTS

Correction of aberrant splicing in HeLa EGFP-654 cells by EEV1-PMO654

We first evaluated the suitability of the EEV platform for *in vitro* delivery of oligonucleotide cargo by utilizing the HeLa EGFP-654 cellular model,[16] which contains a mutated human β-globin intron (IVS2-654) at nucleotide 105 of *EGFP* gene and prevents correct translation of EGFP mRNA. Treatment of HeLa EGFP-654 cells with ASOs, such as PMOs, could switch the splicing and restore expression of EGFP (Figure 1A). Treatment of HeLa EGFP-654 cells with vehicle or unconjugated PMO654 resulted in negligible splice switching, as evidenced by the presence of the 160-bp full-length band and near absence of the corrected 87-bp reverse-transcriptase polymerase chain reaction (RT-PCR) product (Figure S1A). In contrast, EEV1-PMO654 conjugates (Figure 1B) were capable of eliciting splice switching and production of the corrected 87-bp product (Figure S1A). The resulting transcript produced by splice-switching EEV1-PMO654 conjugates was functional and resulted in dose-dependent increases in EGFP expression, as quantified by flow cytometry. The 0.6 µM EEV1-PMO654-treated group produced a fluorescent signal similar to that observed in the 20 µM PMO654 group and an ~3-fold increase in fluorescence between the EEV1-PMO654 and PMO654 treatment groups in a preliminary experiment (Figure S1B). These results were supported by live-cell fluorescence microscopy, which did not show discernable EGFP fluorescence in vehicle- or naked PMO-treated cells, but robust, homogeneous EGFP fluorescence was observed with EEV1-PMO654 conjugate treatment (Figure 1C). Together, these data support that EEV1-PMO654 is effectively internalized and localized in the nucleus to facilitate functional oligonucleotide delivery.

Correction of splicing in EGFP-654 and C57BL/10 mice

To determine whether EEV platform-enhanced PMO delivery efficiency is maintained *in vivo*, we evaluated PMO654 and EEV1-PMO654 conjugates in EGFP-654 mice. All *in vivo* injection doses discussed in this report are represented as the administered EEV1-PMO, in which a consistent molar dose of PMO was administered. First, local tissue delivery efficiency was assessed following 3 consecutive daily intramuscular (i.m.) injections of PMO654 and

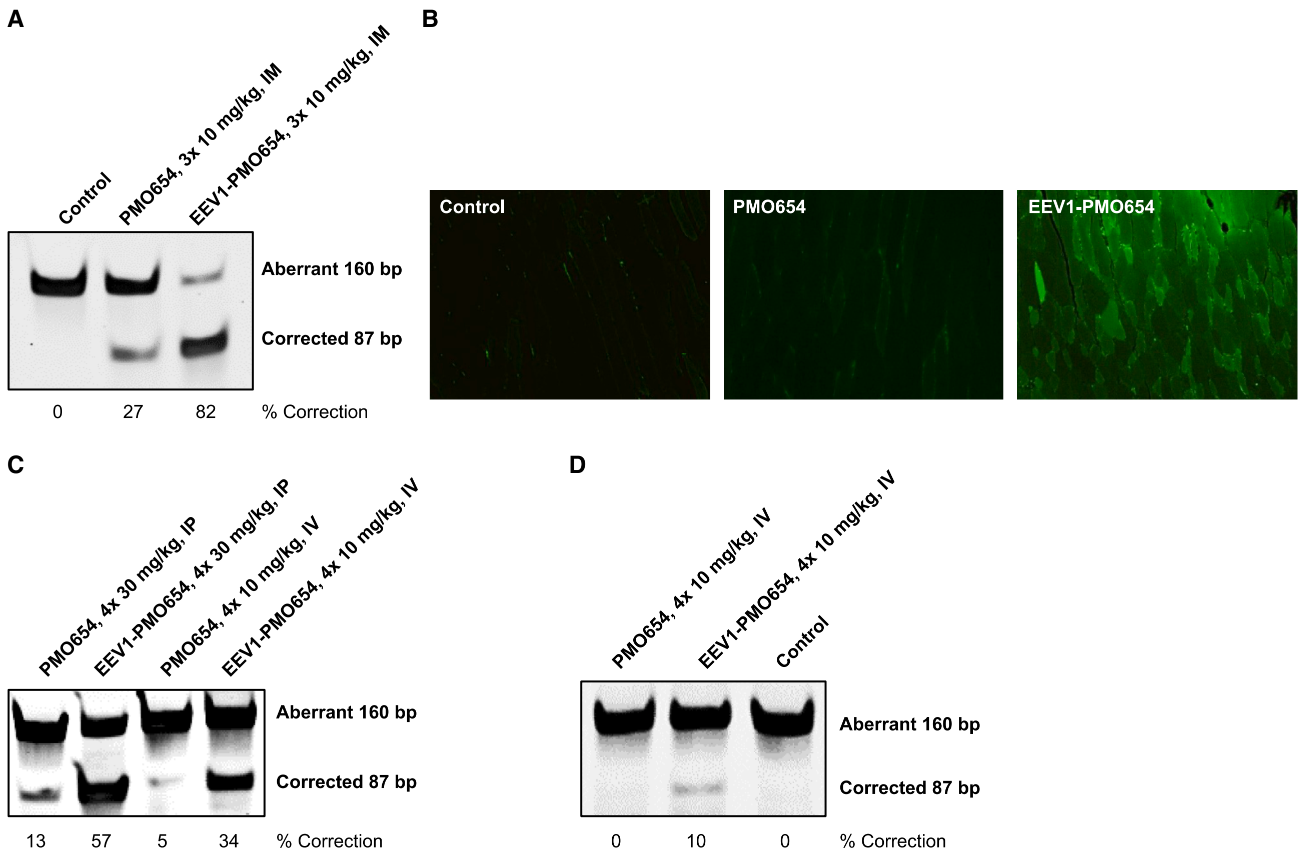

Figure 2. *In vivo* efficacy of unconjugated PMO654 and EEV1-PMO654 conjugates

(A) The exon skipping efficacy following 3 consecutive daily 10 mg/kg i.m. injections in EGFP-654 mice quantified by RT-PCR. (B) Fluorescence microscopy images of muscle sections from EGFP-654 mice treated with unconjugated PMO654 or EEV1-PMO654. (C and D) Splice correction in EGFP-654 mice as quantified by RT-PCR 1 day after i.v. or i.p. injection in the (C) diaphragm and (D) heart at the indicated doses.

EEV1-PMO654. Under these conditions, 3× daily 10 mg/kg unconjugated PMO654 produced much less splice correction than EEV1-PMO654 when evaluated 1 day following the last injection in muscle tissue (quadriceps) adjacent to the injection site (Figure 2A). EEV1-PMO654 treatment produced functional EGFP protein, with diffuse EGFP fluorescence observed in muscle sections taken near the injection site; however, only minimal fluorescence was observed in unconjugated PMO-treated tissue, and no signal was evident in vehicle-treated tissue (Figure 2B). These findings reveal that unconjugated PMOs are incapable of effectively penetrating cells, escaping the endosome, and accessing the nucleus, despite local i.m. injections, which result in high biodistribution.

Additionally, we evaluated splice-switching activity in EGFP-654 mice following intravenous (i.v.) or intraperitoneal (i.p.) injections. Treatment with 4 consecutive daily 10 mg/kg i.v. or 30 mg/kg i.p. doses of unconjugated PMO654 yielded much less corrected transcript in the diaphragm 1 day after the last injection than the EEV1-conjugated PMO654 (Figure 2C). Although the RT-PCR result is a not an absolute quantitative measurement of exon skipping, the results clearly showed a trend of significantly enhanced exon skipping efficacy of PMO after conjugation with an EEV construct. The magnitude of this trend for increased efficacy was maintained in the heart, with 4× PBS (control) and 4× daily 10 mg/kg unconjugated PMO654 found to be below the detection limit for splicing correction compared with ∼10% for 10 mg/kg EEV1-PMO654 when evaluated 1 day after the last injection (Figure 2D).

To ensure that EEV1-enhanced PMO delivery was generalizable and could modulate expression of an endogenous gene, we synthesized EEV1 conjugates of PMO-23 to promote skipping of murine *Dmd* exon 23 and confirmed target engagement in C57BL/10 mice. Successful nuclear delivery of PMO-23 results in production of a *Dmd* transcript-excluding exon 23. Systemic i.v. administration of unconjugated PMO produced low levels of exon 23 skipping across all tissues evaluated, while an equivalent dose of EEV1-PMO-23 yielded robust exon 23 skipping in quadriceps, transversus abdominis, and diaphragm muscles, with modest results observed in the heart (Figure S2). Importantly, EEV1 conjugation significantly enhanced delivery across all tissues evaluated; however, robust efficacy in the heart remains a challenge.

A rational EEV construct design enhances the efficacy of oligonucleotide therapeutics

EEV1 was evaluated in the context of the *mdx* mouse model, a naturally occurring murine model of DMD. This murine model arises from a nonsense mutation in exon 23 of the mouse *Dmd* gene, resulting in little to no production of functional dystrophin protein. Overall, while superior to unconjugated PMO, EEV1-conjugation did not drive significant levels of exon skipping in the heart, which is essential for improving and prolonging the quality of life for DMD patients (Figure S2). Therefore, we investigated ways to further improve cellular uptake, endosomal escape, and nuclear localization. For sterically blocking ASOs, such as PMOs, nuclear localization is a prerequisite for pre-mRNA binding and subsequent activity. To that end, we modified the EEV1 structure to include a cationic nuclear localization sequence (NLS), PKKKRKV, derived from simian virus 40 (SV40), to promote nuclear localization[17] (Figure 3A). This next-generation EEV, termed EEV2, was postulated to possess significantly enhanced nuclear localization, leading to improved splice-switching and exon-skipping activities.

EEV2-PMO654 containing a lissamine rhodamine B label (EEV2-PMO654-lissamine rhodamine B [LSR]) was first evaluated *in vitro* (including EEV1-PMO654-LSR and PMO654-LSR as comparators) in HeLa-EGFP-654 cells. Treatment with 2 μM EEV2-PMO654-LSR resulted in an ∼19-fold increase in EGFP fluorescence relative to unconjugated PMO and a 9-fold increase relative to EEV1-PMO654-LSR, as determined by flow cytometry (Figures S3A) and fluorescence microscopy (Figure 3B). This result was supported by flow cytometry quantification of cellular uptake, which showed similar improvements in whole-cell uptake relative to PMO and EEV1-PMO654 (Figure S3B).

We next evaluated *in vivo* pharmacodynamic efficacy by performing a head-to-head comparison of EEV1- and EEV2-conjugated PMO-23 in *mdx* mice. Consistent with the *in vitro* data, EEV2-PMO-23 demonstrated significantly enhanced exon 23 skipping across all tissues evaluated 7 days following a single 20 mg/kg i.v. injection relative to EEV1-PMO-23 and unconjugated PMO-23 alone (Figure 3C). A major focus of EEV construct optimization was to overcome the challenges associated with cardiac muscle delivery, which EEV2-PMO-23 robustly achieved with ∼50% exon 23 skipping in the heart relative to undetectable levels of skipping for groups treated with EEV1-PMO-23 and unconjugated PMO-23 (Figure 3C).

Systemic administration of a single i.v. dose of EEV2-PMO-23 conjugate results in exon skipping and robust dystrophin production in *mdx* mice

To better understand the therapeutic profile of EEV2-PMO-23 *in vivo*, we evaluated efficacy in *mdx* mice and focused on the magnitude and durability of effect (Figure 4A). The effect of a single i.v. administration of 20 mg/kg EEV2-PMO-23 on exon 23 skipping and dystrophin production was evaluated 1, 2, and 4 weeks post injection to assess the duration of the effect. One week post injection, EEV2-PMO-23 treatment resulted in high exon 23 skipping across skeletal and cardiac muscle, with exon 23 skipping levels of 61% in the heart, 79% in the diaphragm, 74% in the quadriceps, and 71% in the transversus abdominis (Figure 4B). Exon 23 skipping levels persisted for at least 4 weeks, suggesting EEV platform-mediated delivery and accumulation of PMO-23 in the desired tissue with a long residence time. The capability of EEV2-PMO-23 to restore dystrophin production was evaluated by quantification of tissue dystrophin levels via immunodetection after capillary electrophoresis. Note that skipping exon 23 yields a slightly truncated dystrophin that is not distinguishable from the full-length protein. A single i.v. injection of EEV2-PMO-23 restored dystrophin protein expression by ∼11% in the heart, ∼11% in the diaphragm, ∼4% in the quadriceps, and ∼8% in the transversus abdominis (Figure 4B) relative to C57BL/10 wild-type (WT) mice 1 week post injection. As observed with exon 23 skipping, dystrophin restoration persisted and was maintained through 4 weeks in the diaphragm, quadriceps, and transversus abdominis. Consistent with previous observations, a gradual decrease in dystrophin expression was observed over time in the heart following a single dose, although small group sizes limit these conclusions.

Dystrophin protein expression does not necessarily result in restoration of muscle function because proper localization of dystrophin and restoration of the dystrophin-associated protein complex (DAPC) is required.[18] To assess proper localization, we evaluated tissue samples collected 4 weeks following the single i.v. dose of dystrophin by immunohistochemistry (Figure 4C). Age- and strain-matched WT mice showed strong membrane-associated dystrophin staining, while saline-treated *mdx* mice expressed little to no dystrophin across multiple muscle tissue, as anticipated. Treatment with EEV2-PMO-23 resulted in positive dystrophin staining across all tissues evaluated and showed proper dystrophin membrane localization. Importantly, dystrophin expression was observed throughout each individual tissue, indicating robust EEV platform-mediated tissue penetration and internalization in dense, multicellular environments.

Systemic administration of multiple i.v. doses of EEV2-PMO-23 conjugate yields significant exon skipping, dystrophin production, and biomarker correction in *mdx* mice

To evaluate whether multiple injections of EEV2-PMO-23 maintained or improved exon 23 skipping and the resultant dystrophin expression, we delivered once-weekly i.v. doses of 10 mg/kg compound for 4 weeks (Figure 5A). Tissue assessed 1 week after the fourth injection showed significant exon 23 skipping (>30% in the heart and >50% in skeletal muscles) and dystrophin protein restoration (>30% in all tissues evaluated) (Figure 5B). Conversely, weekly i.v. dosing with 10 mg/kg unconjugated PMO-23 only resulted in minimal exon skipping and undetectable dystrophin protein levels across all tissues evaluated at the same time point. The robust efficacy of EEV2-PMO-23 for dystrophin restoration was also demonstrated by immunofluorescence staining, in which dystrophin expression was observed to be uniformly distributed in the heart and localized in the cell membrane (Figure 5C). Furthermore, repeated treatment with EEV2-PMO-23 dramatically reduced serum creatine kinase (a standard biomarker for muscle damage) in *mdx* mice down to the range observed in WT mice, indicating restored integrity of the

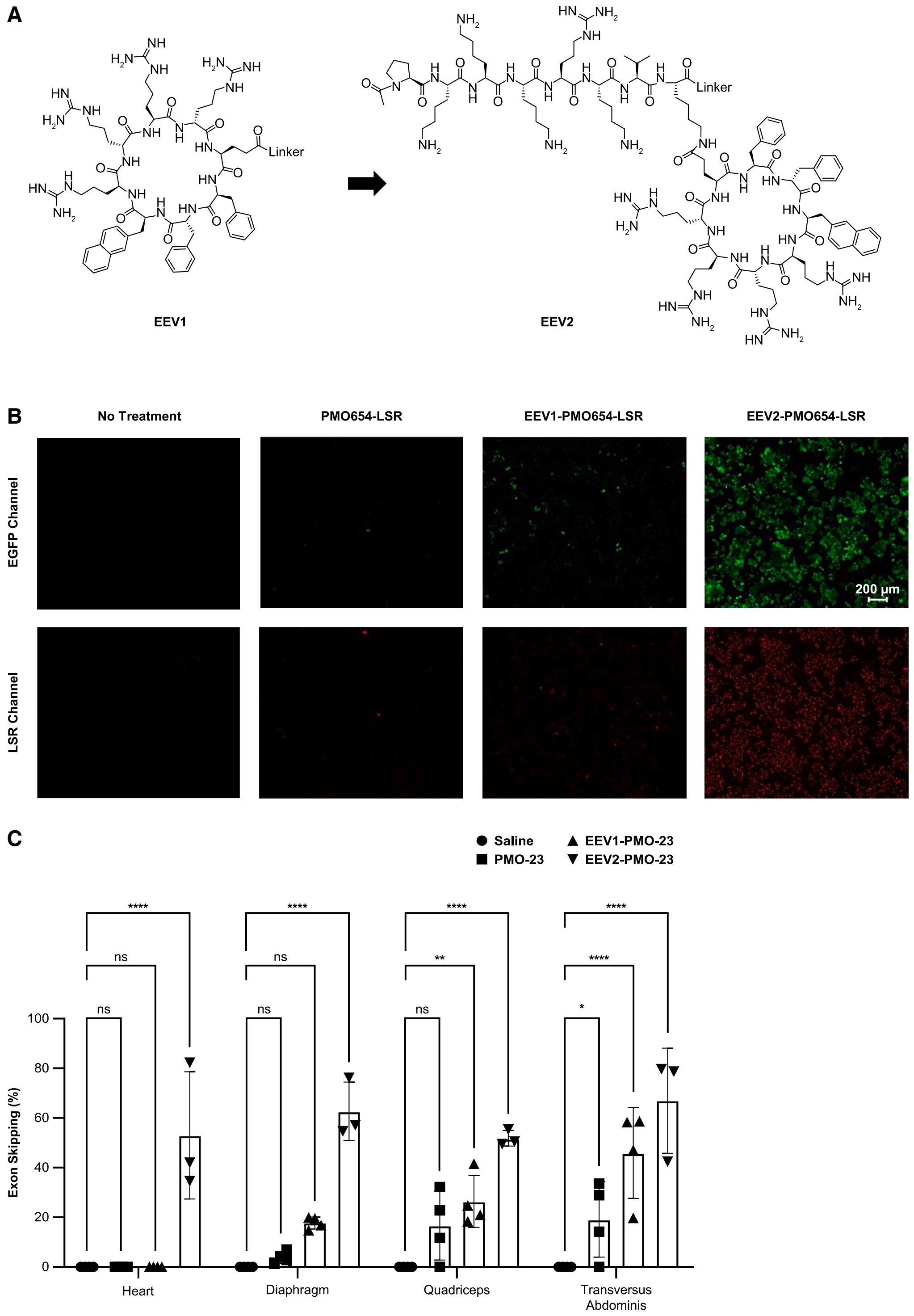

Figure 3. Design of second-generation EEV2-PMO-23 and comparison of *in vitro* and *in vivo* efficacy with first-generation EEV1-PMO-23 conjugates
(A) Structure and design of the second-generation EEV2-PMO-23. (B) Fluorescence images of restored EGFP expression in HeLa *EGFP-654* cells. 2 μM unconjugated PMO, EEV1-PMO654 (2 μM PMO equivalent), and EEV2-PMO654 (2 μM PMO equivalent) were labeled with lissamine rhodamine B (LSR). (C) *In vivo* efficacy of EEV-PMO-23 conjugates in *mdx* mice after i.v. administration, as determined by exon skipping (mean ± standard deviation) measured by RT-PCR. ****$p < 0.0001$; ***$p < 0.0005$; **$p < 0.005$; *$p < 0.05$; ns, not significant as assessed by two-way ANOVA and Dunnett multiple-comparisons test compared with vehicle.

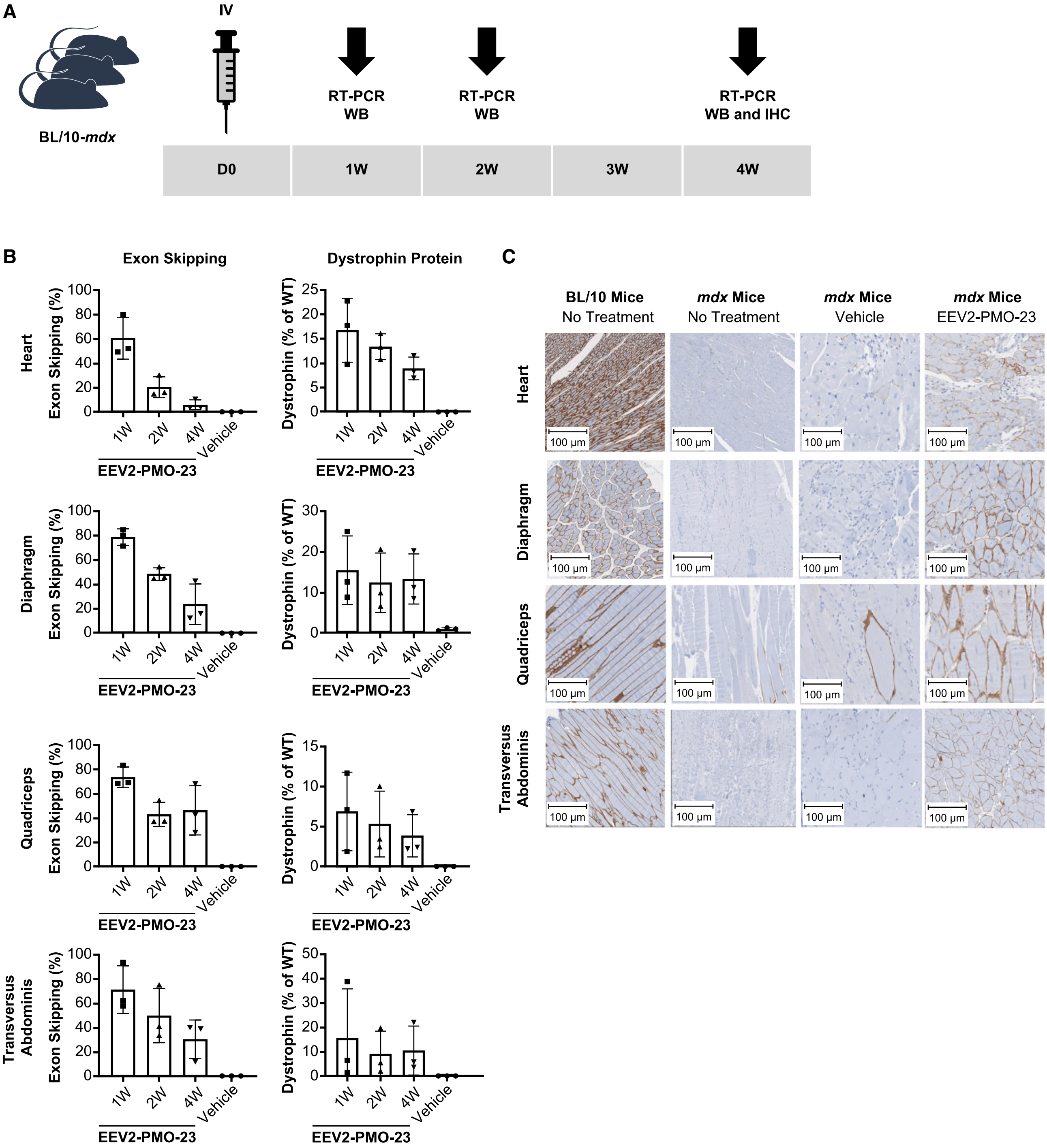

Figure 4. Pharmacodynamic effects of EEV2-PMO-23 in *mdx* mice after single i.v. injection at 20 mg/kg

(A) The study design to evaluate the duration of effect of EEV-PMO-23 up to 4 weeks. (B) Exon 23 skipping efficacy (mean ± standard deviation) was analyzed by RT-PCR, and dystrophin restoration was quantified by immunodetection after capillary electrophoresis and presented relative to WT dystrophin levels. Data presented as mean ± standard deviation. (C) Immunohistochemistry (IHC) staining images for dystrophin in various muscle tissues 4 weeks post injection.

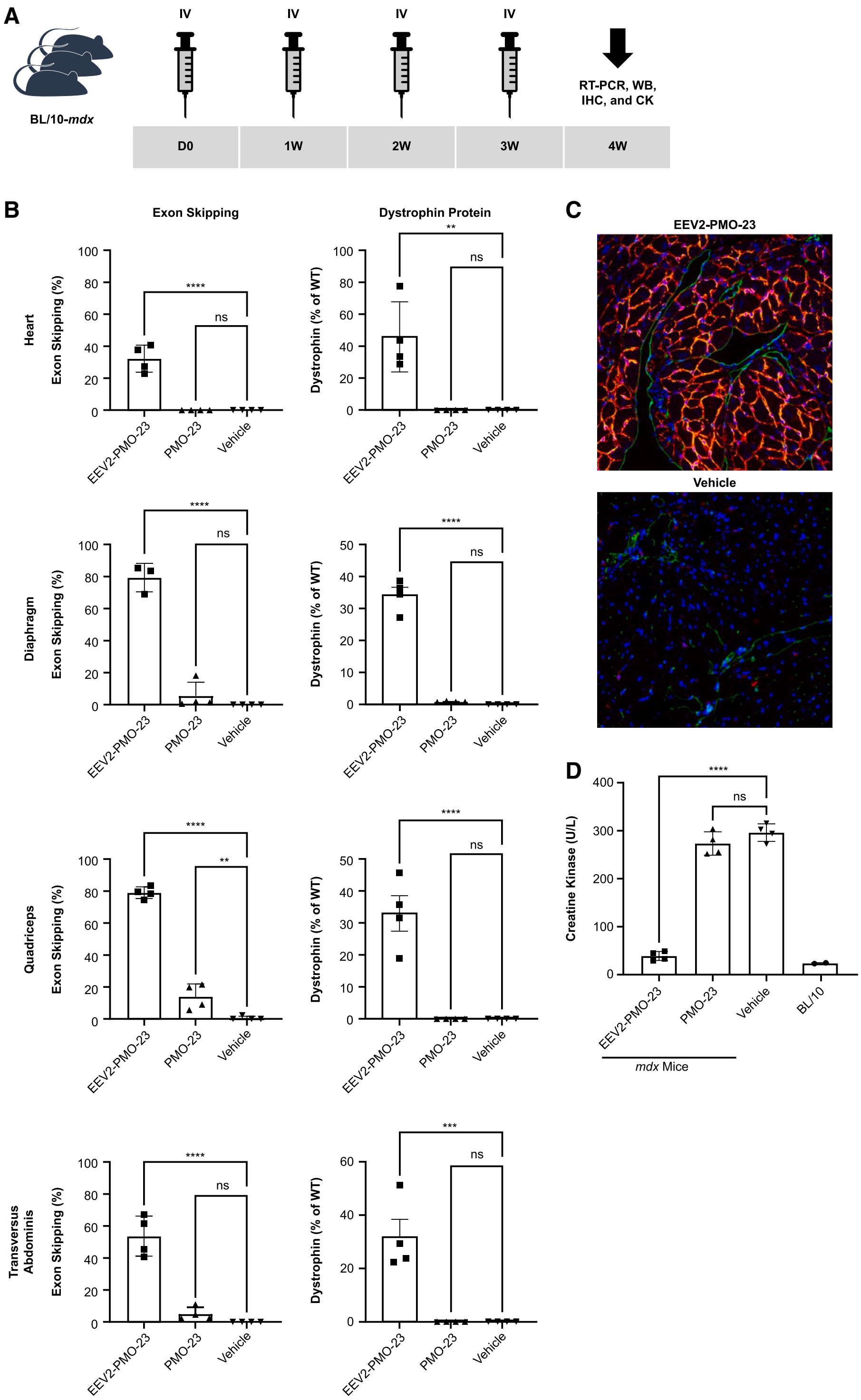

Figure 5. Pharmacodynamic effects of EEV2-PMO-23 in *mdx* mice 1 week after 4 once-weekly doses of 10 mg/kg

(A) Study design for repeated dosing. (B) Exon 23 skipping efficacy (mean ± standard deviation) was analyzed by RT-PCR, and dystrophin restoration was quantified by immunodetection after capillary electrophoresis and plotted relative to WT animals. (C) IHC staining of cardiac tissues: DAPI (blue), endothelial cells using anti-CD31 (green), vimentin-stromal (yellow), and dystrophin (red). The image is about 80 μm in width. (D) Serum creatine kinase (CK) levels after measured after treatment. ****p < 0.0001, ***p < 0.0005, **p < 0.005, *p < 0.05 as assessed by one-way ANOVA and Dunnett's multiple-comparisons test compared with vehicle.

muscle membrane and resolution of muscle damage; contrastingly, unconjugated PMO-23 had no effect (Figure 5D).

EEV platform conjugation enhances PMO efficacy in cellular models of FSHD

Because EEV platform conjugation was capable of robustly improving PMO-induced splicing modulation in EGFP and DMD preclinical models, we further investigated whether EEV-PMO conjugates could be applied to a second neuromuscular indication, FSHD. FSHD results from aberrant expression of *DUX4*, a transcription activator for regulating RNA expression that plays a critical role in embryonic development, which is normally silenced between the 4- and 8-cell stage of embryonic development.[19] There are no approved therapies for FSHD; however, reduction of DUX4 expression is a promising strategy for FSHD.[20–22] We employed a PMO that targets the polyadenylation signal (PAS) region (PMO-PAS) of the *DUX4* transcript to prevent DUX4 expression and conjugated it to EEV2. Treatment of FSHD patient-derived lymphoblastoid cells with EEV2-PMO-PAS resulted in significant, dose-dependent reductions in the DUX4 downstream target genes *MBD3L2* (methyl-CpG binding domain protein 3-like 2), *ZSCAN4* (zinc-finger and SCAN domain-containing 4), and *TRIM43* (tripartite motif-containing protein 43) levels (Figure 6A). Further evaluation of EEV2-PMO-PAS in FSHD patient-derived muscle cells showed a significant reduction in *ZSCAN4* transcript expression to near-healthy levels (Figure 6B).

DISCUSSION

For the first time, we show that next-generation EEV platform conjugates can serve as a platform to enable intracellular delivery of biological agents *in vitro* and *in vivo*. We demonstrated the potential of this EEV technology for delivery of ASOs and evaluated the EEV-oligonucleotide conjugates in 2 distinct preclinical models of neuromuscular disorders. Single-stranded, ASO-based therapeutic agents have been studied in a broad range of disease areas and can utilize several mechanisms of action to exert a therapeutic effect on RNA.[23] Despite their promise, unconjugated ASO therapeutics are still associated with limited bioavailability to the target tissue and poor translocation across the plasma membrane.[24] Other strategies for ASO delivery, such as conjugation with a receptor-targeting antibody,[25] can enhance ASO localization in the target tissue but do not improve the poor endosomal escape efficiency of ASOs. Conjugation of CPPs to ASOs has improved these limitations, and although CPPs provide increased cellular uptake, there is room for enhanced delivery to target sequences within cells and organs. Here, we demonstrate that addition of a cyclic CPP-derived EEV enhances intracellular delivery of a PMO compared with unconjugated PMO. Covalent conjugation of a splice-switching PMO (PMO654) with a first-generation EEV construct resulted in dose-dependent enhancement of PMO splice switching and a subsequent increase in EGFP expression by improving cellular uptake. Furthermore, EEV1-PMO654 conjugates demonstrated *in vivo* activity following systemic or local administration in EGFP-654 transgenic mice. Across skeletal and cardiac muscle, 2 sites of pathophysiology in neuromuscular disorders, EEV platform conjugation improved PMO-induced splice switching by more than 5-fold. We further demonstrated that EEV1-PMO-23 elicited improved exon 23 skipping across multiple tissues in C57BL/10 mice relative to unconjugated PMO, which had only minimal efficacy. Taken together, these findings support generalizable delivery of PMO cargo using EEV1.

We next assessed the ability of the EEV platform to improve PMO-mediated exon skipping in skeletal and cardiac muscle of *mdx* mice. Currently approved exon-skipping therapies for DMD are unconjugated PMOs designed to restore the reading frame and result in production of internally truncated but functional dystrophin protein.[26–29] However, these therapies are associated with low levels of dystrophin protein restoration, especially in cardiac muscle.[30,31] In our study, first-generation EEV1-PMO-23 conjugates displayed moderate exon skipping in skeletal muscle and limited activity in cardiac tissue. To achieve a more potent response through optimization of cellular uptake, an exocyclic, polycationic NLS was incorporated to yield a highly active next-generation EEV construct (EEV2). *In vitro* uptake of EEV2-PMO654 in HeLa EGFP-654 cells resulted in more than a 10-fold improvement in EGFP expression relative to EEV1-PMO654 (Figure S3). The increase in efficacy demonstrated by EEV2-PMO conjugates also carried over to *in vivo* studies, where we observed greater than 15% and greater than 60% exon 23 skipping in cardiac and skeletal muscle, respectively, 7 days after the single i.v. 20 mg/kg dose. Exon skipping was durable for up to at least 4 weeks after the single injection. Additionally, repeat administration of a lower 10 mg/kg i.v. dose resulted in greater than 30% dystrophin protein restoration in the heart 7 days post injection, and serum creatine kinase was reduced to WT levels. The advantages of this lower dose were substantial exon skipping and dystrophin protein restoration in cardiac muscle and less drug accumulation in the liver and kidneys.

Restoration of dystrophin function is critical to prevent the progressive muscle weakness, respiratory insufficiency, and cardiomyopathy seen in patients with DMD. Importantly, restoration of dystrophin proteins in the *mdx* mouse model occurred in skeletal and cardiac muscle, showing promise for cardiac delivery using a modality for which extensive cardiac exposure remains a challenge. Furthermore, elevated creatine kinase levels returned to baseline following EEV2-PMO treatment, indicating improvements in muscle integrity. This improved muscle integrity is supported by immunofluorescence results displaying exceptional dystrophin restoration and proper localization along the membrane across different muscle tissues. These results suggest that our next-generation EEV-PMO conjugates are a major improvement over previous intracellular delivery systems and unconjugated PMOs.

In addition to examining exon-skipping oligonucleotides in DMD models, we also demonstrate conjugation of our EEV technology to sterically blocking oligonucleotides specific for FSHD. In FSHD, aberrant overexpression of the fetal *DUX4* gene in skeletal muscle is the root cause of disease pathophysiology.[32] To determine whether our EEV-PMO approach was able to disrupt polyadenylation to destabilize *DUX4* transcripts and subsequently reduce pathogenic

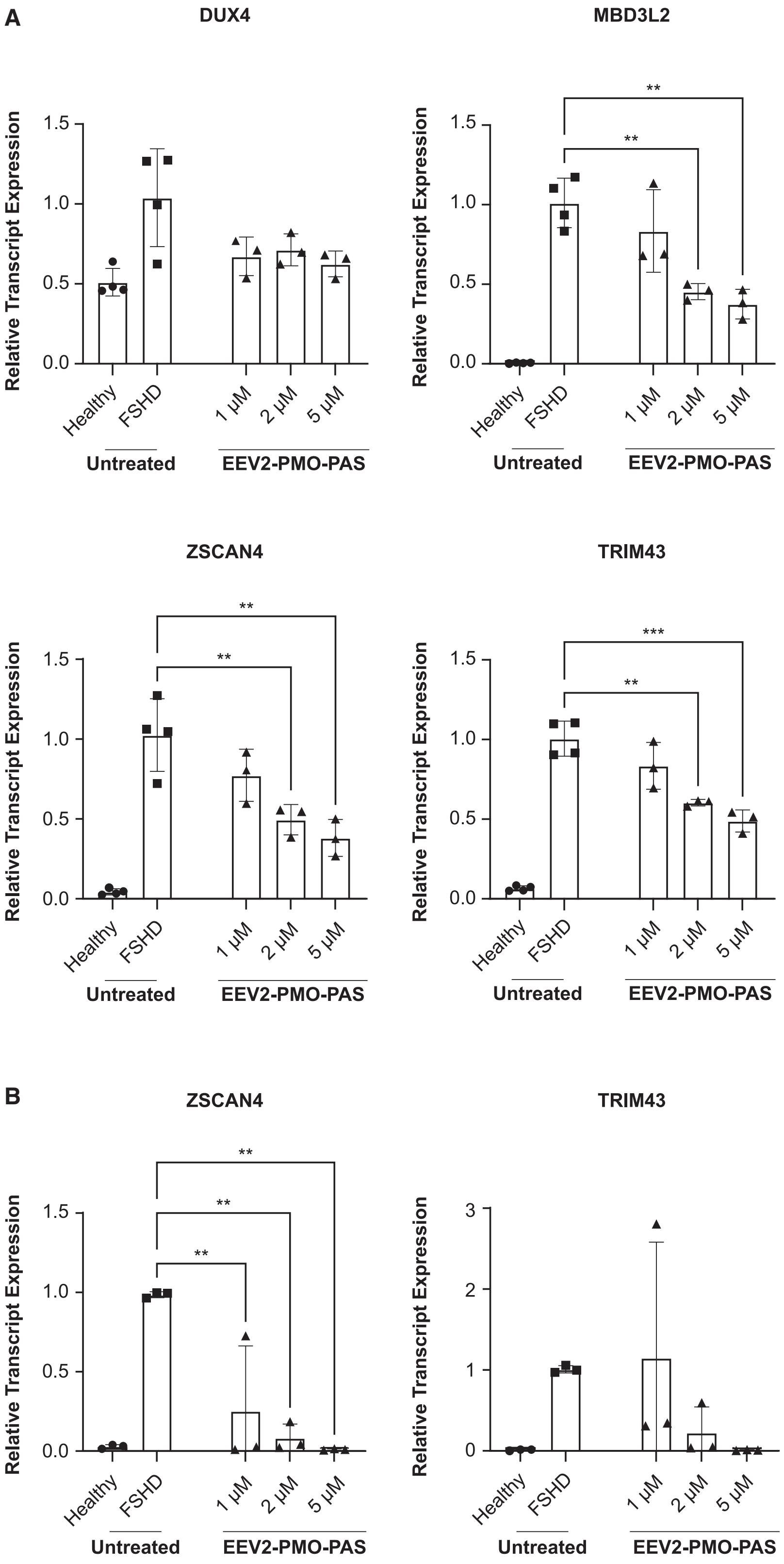

Figure 6. Efficacy of EEV2-PMO conjugates in FSHD *in vitro* models

(A) mRNA expression levels in lymphoblastoid (LB) cells after treatment with EEV2-PMO-PAS for *DUX4* and downstream targets as determined by RT-PCR. (B) mRNA expression levels in FSHD patient-derived muscle cells after treatment with EEV2-PMO-PAS for the *DUX4* downstream transcription targets ZSCAN4 and TRIM43 as determined by RT-PCR. ***$p < 0.0005$, **$p < 0.005$ as assessed by one-way ANOVA and Dunnett's multiple-comparisons test compared with untreated FSHD cells.

downstream expression of DUX4 targets, we developed an FSHD-specific EEV-PMO conjugate (EEV2-PMO-PAS). The observed reduction of the *DUX4* transcript and its downstream targets after treatment with EEV2-PMO-PAS further supports the applicability of our EEV platform to multiple neuromuscular disorders.

These results demonstrate the significant therapeutic potential of our EEV-oligonucleotides to upregulate and downregulate gene expression neuromuscular diseases because EEV platform conjugates robustly deliver covalently conjugated oligonucleotides to skeletal and cardiac muscle cells across 3 preclinical models. We are further examining the broad applicability of this EEV platform in other disease areas through high-throughput library screening *in vitro*. This approach allows customization of the platform for optimal intracellular uptake and efficient endosomal escape in several tissue types. Moreover, this EEV platform has the flexibility to be conjugated to a variety of biological agents and therefore has the potential to address unmet medical needs by reaching previously undruggable therapeutic targets.

MATERIALS AND METHODS

Preparation of EEV-PMO conjugates

All PMOs were obtained from Gene Tools (Philomath, OR, USA). For experiments using unconjugated ASOs, PMOs were purified using reverse-phase high-performance liquid chromatography (HPLC). EEV1 and EEV2 peptides were synthesized and purified at WuXi Chemistry Service Unit using standard solid-phase peptide synthesis (SPPS). EEV-PMO conjugates were prepared through either amide-bond formation or copper-free azide-alkyne cycloaddition. For amide bond formation, a peptide with free acid was pre-activated by reacting it with hexafluorophosphate azabenzotriazole tetramethyl uronium (2.0 equivalent) and *N,N*-diisopropylethylamine (2.0 equivalent) in DMSO (10 mM). After 10 min at room temperature, the pre-activated solution was combined with a solution of PMO with 3′ secondary amine in DMSO (10 mM) and mixed for 2 h at room temperature. The reaction was monitored by LC-mass spectrometry (MS). Upon completion, the product was purified using reverse-phase chromatography with C18 columns on HPLC, followed by salt exchange. For preparation of EEV-PMO through strain-promoted azide-alkyne cycloaddition chemistry, EEV-azide (1.5 equivalent) was added to a solution of PMO-3′-cyclooctyne in water (5–10 mM) and mixed thoroughly. The reaction was incubated for approximately 12 h at room temperature and monitored by LC-MS. Upon completion, the product was purified using reverse-phase chromatography followed by salt exchange.

In vitro studies with HeLa-EGFP-654 cells

HeLa-EGFP-654 cell line

The HeLa-EGFP-654 model is constructed by inserting a cryptic splice site utilizing a mutation derived from human β-globin into *EGFP* intron 2, resulting in aberrant pre-mRNA splicing and arrested EGFP expression (Figure 1A).[33] Steric blocking of this cryptic splice site with a splice-switching ASO results in splicing correction and EGFP expression. HeLa-EGFP-654 cells (LCCC Tissue Culture Facility, The University of North Carolina at Chapel Hill, Chapel Hill, NC, USA) were maintained in F-12K medium (30-2004; American Type Culture Collection, Manassas, VA, USA) supplemented with 10% fetal bovine serum (FBS) at 37°C, 5% CO_2. Cells were plated at a density of 10^5 cells/well in 24-well plates and cultured overnight. The following day, the growth medium was removed, and HeLa-EGFP-654 cells were treated with PMO or EEV-PMO conjugates diluted to the indicated concentrations in fresh growth medium, respectively. The cells were analyzed 24 or 36 h after treatment for splicing analysis, and EGFP fluorescence was evaluated by fluorescence microscopy and flow cytometry.

Splicing correction by RT-PCR

To determine splicing correction, treated HeLa-EGFP-654 cells were lysed in TRI reagent (AM9738; Thermo Fisher Scientific, Waltham, MA, USA) for total RNA isolation, and 1 μg RNA was used for RT-PCR using the OneTaq RT-PCR Kit (New England Biolabs, Ipswich, MA, USA) according to the manufacturer's protocol. Reverse transcription was carried out at 42°C for 1 h, and 1 μL cDNA was used for the PCR reaction with the following program: 1 cycle, 95°C, 30 s; 20 cycles, 95°C, 30 s, 58°C, 30 s, 68°C, 30 s; 1 cycle, 68°C, 5 min. The PCR products were analyzed by 4% E-gel (Thermo Fisher Scientific).

Fluorescence analysis

To determine the EGFP restoration and lissamine rhodamine by flow cytometry analysis, treated HeLa-EGFP-654 cells were dissociated with 1× Gibco trypsin-EDTA (25200072; Gibco, Thermo Fisher Scientific) and resuspended in a solution containing 1× PBS, 2% FBS, and 2 μg/mL propidium iodide. Approximately 10,000 cells of each sample were subjected to flow cytometry analysis by BD LSRFortessa (BD Biosciences, Franklin Lakes, NJ, USA; Figure S1) and 2,000 cells by BD FACSCanto (BD Biosciences; Figure S3). Dead cells were omitted by gating of side versus forward scatter, and histograms of cell number versus fluorescence intensity were generated and analyzed with FlowJo (BD Biosciences). Fluorescence microscopy of treated HeLa-EGFP-654 cells was performed with a customized inverted fluorescence microscope using the GFP channel.

In vivo mouse models and studies

Animal studies

All mouse studies were conducted at Tufts University (Boston, MA, USA), SmartLabs (Boston, MA, USA), or Dartmouth College (Hanover, NH, USA) in compliance with approved institutional animal care and use committee guidelines. The housing temperature was set to 20°C–23°C with approximately 50% relative humidity and a 12-h light/dark cycle. Food and water were provided *ad libitum*. Animals were acclimated to handling and testing procedures prior to functional testing.

EGFP-654 mice

The EGFP-654 transgenic mouse model (027617; The Jackson Laboratory, Bar Harbor, ME, USA) contains an EGFP gene interrupted by human β-globin intron 2 with mutated nt654.[16] PMO654 (5′-GCT ATTACCTTAACCCAG-3′) and EEV1-PMO654 were delivered to

EGFP-654 mice by 4× daily i.p., 4× daily i.v., or 3× daily i.m. injections. Mice were euthanized 1 day after the last injection, and tissues were collected for downstream analyses. Tissue from mice treated by i.m. injections were imaged to probe for GFP fluorescence.

mdx *mice*

The *mdx* mouse is a DMD mouse model with a nonsense mutation in *Dmd* exon 23.[34] PMO-23 (5′-GGCCAAACCTCGGCTTACCTGA AAT-3′), EEV1-PMO-23, and EEV2-PMO-23 were delivered to *mdx* mice by i.v. injection with the indicated dosing regimen. Mice were 6–9 weeks old at the start of each experiment. The mice were euthanized 7 days after the last injection, and tissues were collected for RT-PCR, dystrophin protein quantification via ProteinSimple (Minneapolis, MN, USA) Jess capillary electrophoresis, and immunostaining analysis.

C57BL/10 mice

PMO-23 and EEV1-PMO-23 were delivered to C57BL/10 mice by i.v. lateral tail vein injection. The mice were euthanized 7 days after the last injection, and tissues were collected for RNA isolation and RT-PCR analysis.

Splicing analysis by RT-PCR in EGFP-654 mouse tissues

Mouse tissue from EGFP-654 mice injected via i.p., i.v., and i.m. routes were analyzed by RT-PCR following a similar protocol as described for the splicing correction analysis of HeLa-EGFP654 cells. Briefly, the tissues were lysed in TRI reagent (AM9738; Thermo Fisher Scientific) for total RNA isolation according to the manufacturer's instructions. Reverse transcription was carried out at 42°C for 1 h, and 1 μL cDNA was used for PCR with the following protocol: 1 cycle, 95°C, 30 s; 20 cycles, 95°C, 30 s, 58°C, 30 s, 68°C, 30 s; 1 cycle, 68°C, 5 min. PCR products were analyzed by 2% agarose gel. Product bands were quantified by densitometry analysis with ImageJ software, and skipping efficacy was calculated with the following equation: skipping (%) = A/(A + B) × 100, where A is the intensity of the skipped band, and B is the intensity of the full-length band.

Exon 23 skipping analysis by RT-PCR in mdx *and C57BL/10 mice*

RNA was extracted from muscle tissue using the RNeasy Mini Kit (74106; QIAGEN, , Hilden, Germany) according to the manufacturer's protocol. Nested RT-PCR was performed with 200 ng of total RNA for primary amplification using the One-step RT-PCR Kit (210212, QIAGEN). A reaction solution of 50 μL was prepared according to the manufacturer's protocol. Secondary amplification was performed with 1 μL of PCR product from the primary amplification in a 50-μL reaction using OneTaq Hot Start 2× Master Mix (M0484L, New England Biolabs) following the manufacturer's protocol. The PCR programs for primary RT-PCR and secondary PCR were as follows: for primary amplification, 1 cycle, 50°C, 30 min; 1 cycle, 95°C, 15 min; 30 cycles, 95°C, 30 s, 58°C, 1 min, 72°C, 2 min; 1 cycle, 72°C, 10 min and for secondary amplification, 1 cycle, 95°C, 1 min; 20 cycles, 95°C, 30 s, 55°C, 1 min, 72°C, 2 min; 1 cycle, 72°C, 5 min. The forward and reverse primers for primary amplification were 5′-CAGAATTCTGCCAATTGCTGAG-3′ and 5′-TTCTT CAGCTTGTGTCATCC-3′, respectively. For secondary amplification, the forward and reverse primers were 5′-CCCAGTCTACCA CCCTATCAGAGC -3′ and 5′-CCTGCCTTTAAGGCTTCCTT-3′, respectively. PCR products were analyzed by E-gel (Thermo Fisher Scientific). PCR products were quantified by densitometry using ImageJ software, and the skipping efficiency was calculated with the following equation: skipping (%) = A/(A + B) × 100, where A is the intensity of the band with exon skipping, and B is the intensity of the full-length band.

Dystrophin protein quantification from mdx *and C57BL/10 mice*

Protein was extracted from muscle tissues using mechanical bead rupture in lysis buffer consisting of 9% SDS, 75 mM Tris (pH 7), 5% 2-mercaptoethanol, and HALT protease inhibitors (78425, Thermo Fisher Scientific) and normalized by tissue mass. ProteinSimple Jess was used for capillary electrophoresis along with immunodetection using anti-dystrophin (Ab154168, 1:1,000; Abcam, Cambridge, UK) and anti-αactinin (MAB8279, 1:100; R&D Systems, Minneapolis, MN, USA). A standard curve was included for each experiment to allow quantification, which consisted of ratios of C57BL/10 murine lysate diluted with *mdx* murine lysate from the respective muscle tissue. ProteinSimple Jess reagents were used as follows and according to the manufacturer's protocols: 66- 440-kDa separation module (SM-W008); dystrophin was detected using the antirabbit detection module (DM-001 or 043-426 when paired with DM-002 for α-actinin detection), and α-actinin was detected with either the anti-mouse near-infrared detection module (DM-009) or the anti-mouse detection module (DM-002). A Gaussian fit was used for each peak for quantification, and the ratio of dystrophin to α-actinin was interpolated to the standard curve for each experiment.

Creatine kinase assay

Quantification of serum creatine kinase activity was performed with a commercial kit (MAK116; Sigma-Aldrich, St. Louis, MO, USA) according to the manufacturer's instructions.

Immunostaining

7-μm-thick sections of flash-frozen tissue were prepared using a Leica CM 1950 cryostat. For immunofluorescence analysis, sections were rehydrated by incubating with 1× phosphate-buffered saline (PBS) 3 times for 5 min. Next, sections were blocked (10% normal goat serum in PBS) for 60 min at room temperature. Primary antibodies were diluted 1:50 in an antibody dilution buffer (10% NGS, 0.3% Triton X-100 in PBS), and sections were incubated overnight at 4°C. Slides were washed 3 times for 5 min with PBS and incubated with secondary antibody, both diluted 1:4,000, for 60 min at room temperature and protected from light. Following dystrophin staining, the slides were counterstained with hematoxylin to visualize nuclei. Slides were washed 3 times for 5 min with 1× PBS protected from light. Sections were mounted with Gold antifade mounting medium and cured for 4 h before imaging. Tissue was embedded into FFPE blocks, and 5-μm-thick tissue sections were prepared using a Leica CM 1950 cryostat. Blocks were deparaffinized using a Ventana Discovery wash (Roche Diagnostics, Indianapolis, IN, USA) for 24 min at 70°C, followed by incubation with

peroxidase-blocking solution for 12 min at room temperature. Immunohistochemistry was performed by warming slides to 37°C and incubating with rabbit anti-dystrophin monoclonal antibody (mAb; ab218198) at a 1:500 dilution for 60 min. Images were acquired using Ventana Discovery Ultra, and image analysis was performed using Indica Labs Halo v.1.7 software.

Cell culture and treatment of FSHD patient-derived cells

Immortalized myoblasts from an FSHD donor (AB1080) and an unaffected individual (KM1421) were obtained from the Institut de Myologie, France, in accordance with French legislation. FSHD is associated with contractions of the D4Z4 repeat in the subtelomere of chromosome 4q. FSHD patient-derived myoblasts were cultured in growth medium consisting of skeletal muscle cell growth medium (PromoCell, Heidelberg, Germany), 2% horse serum (Gibco), 1% chick embryo extract (US Biological, Salem MA, USA), and 0.5 mg/mL penicillin/streptomycin (Gibco). For myogenic differentiation, confluent cultures were switched to differentiation medium consisting of DMEM supplemented with 2% horse serum for 5 days. For cellular treatments, EEV2-PMO-PAS was diluted in differentiation medium and added to confluent myoblasts at the onset of myogenic differentiation. Myoblasts were exposed to EEV2-PMO-PAS throughout differentiation and harvested after 5 days. One FSHD patient-derived lymphoblastoid cell line (GM16283) and 2 lymphoblastoid cell lines from healthy donors (GM16281 and GM16275) were purchased from the Coriell Institute for Medical Research (Camden, NJ, USA) and cultured with RPMI 1640 medium (Gibco) supplemented with 15% FBS at 37°C and 5% CO_2. Cells (2×10^5 cell/mL) were plated in 12-well cell culture dishes, and 1–10 μM of EEV2-PMO-PAS was added to the culture medium 24 h later. Cells were lysed, and total RNA was extracted using QIAcube (QIAGEN) following 24 h of drug exposure.

Biomarker analysis for FSHD

Total RNA was isolated with the QIAGEN RNeasy Mini Kit according to the manufacturer's instructions. For relative quantification of DUX4 target genes, 500 ng RNA was reverse transcribed with the SuperScript IV first-strand synthesis system (Invitrogen) in a 20-μL reaction. Two microliters of cDNA were used for qPCR (SYBR Green Master Mix, Applied Biosystems, Waltham, MA) with gene-specific primers. For FSHD patient-derived lymphoblastoid cells, 1 mg of RNA was used for cDNA synthesis with the High-Capacity cDNA Reverse Transcription Kit (Applied Biosystems) according to the manufacturer's instructions. qRT-PCR was performed with 2 technical replicates per sample using iTaq Universal SYBR Green Supermix (Bio-Rad, Hercules, CA, USA) and gene-specific primer pairs with a QuantStudio 5 real-time PCR system (Thermo Fisher Scientific).

Statistical analysis

Where indicated, statistical significance was assessed by one-way or two-way ANOVA and Dunnett multiple-comparisons test compared with vehicle (Figures 3B, 5B, and 5D) or compared with untreated FSHD cells (Figures 6A and 6B).

DATA AND CODE AVAILABILITY

All therapeutic agents utilized in this study are proprietary to Entrada Therapeutics (Boston, MA, USA). The data that support the findings of our studies are available from the corresponding author upon reasonable request.

SUPPLEMENTAL INFORMATION

Supplemental information can be found online at https://doi.org/10.1016/j.omtn.2023.06.022.

ACKNOWLEDGMENTS

This research was funded by Entrada Therapeutics. Copyediting and studio support for this manuscript was provided by Ashfield MedComms, an Inizio company, and funded by Entrada Therapeutics. Components of the graphical abstract were created with BioRender. We would like to thank Yajun Huang and Wang Li from WuXi AppTec for synthetic chemistry support, Aji Nair (Entrada Therapeutics) for editorial support, and the *in vivo* pharmacology team at Entrada Therapeutics for assisting with data collection.

AUTHOR CONTRIBUTIONS

X.L., M.K., P.G.D., and Z.Q. designed the experiments and wrote the manuscript. N.S. and Z.Q. provided experimental and strategic guidance. M.K. and E.S. designed and synthesized EEV-PMO conjugates. X.L., X.S., and A.P. conducted the *in vitro* studies with Hela-EGFP-654 cells. X.L., C.d.G., and A.B.G. conducted the *in vivo* studies with EGFP-654 mice. X.L., K.J.K., M.D., S.P., and A.P. conducted the *in vivo* studies with C57BL/10 and *mdx* mice. X.S., N.L.E., S.L.B., and M.G. conducted the *in vitro* studies with FSHD patient-derived cells. All authors contributed to the writing of this article, reviewed and revised this article for intellectual content, and approved the final draft for submission. S.P. was an employee of Entrada Therapeutics when this research was conducted.

DECLARATION OF INTERESTS

X.L., M.K., P.G.D., K.J.K., X.S., N.L.E., S.P., A.P., S.L.B., E.S., M.D., M.G., N.S., and Z.Q. are employees of and hold stock in Entrada Therapeutics.

REFERENCES

1. DrugBank Online (2022). Antisense Oligonucleotides. https://go.drugbank.com/categories/DBCAT001709.

2. Migliorati, J.M., Liu, S., Liu, A., Gogate, A., Nair, S., Bahal, R., Rasmussen, T.P., Manautou, J.E., and Zhong, X.-b. (2022). Absorption, distribution, metabolism, and excretion of US Food and Drug Administration–Approved antisense oligonucleotide drugs. Drug Metab. Dispos. *50*, 888–897.

3. Aartsma-Rus, A., Straub, V., Hemmings, R., Haas, M., Schlosser-Weber, G., Stoyanova-Beninska, V., Mercuri, E., Muntoni, F., Sepodes, B., Vroom, E., and Balabanov, P. (2017). Development of exon skipping therapies for Duchenne muscular dystrophy: a critical review and a perspective on the outstanding issues. Nucleic Acid Therapeut. *27*, 251–259.

4. Moulton, H.M., and Moulton, J.D. (2017). Morpholino Oligomers (Springer Science+Business Media LLC).

5. Chen, J.C., King, O.D., Zhang, Y., Clayton, N.P., Spencer, C., Wentworth, B.M., Emerson, C.P., Jr., and Wagner, K.R. (2016). Morpholino-mediated knockdown of

DUX4 toward facioscapulohumeral muscular dystrophy therapeutics. Mol. Ther. *24*, 1405–1411.

6. Lu-Nguyen, N., Malerba, A., Herath, S., Dickson, G., and Popplewell, L. (2021). Systemic antisense therapeutics inhibiting DUX4 expression ameliorates FSHD-like pathology in an FSHD mouse model. Hum. Mol. Genet. *30*, 1398–1412.
7. Heemskerk, H., de Winter, C., van Kuik, P., Heuvelmans, N., Sabatelli, P., Rimessi, P., Braghetta, P., van Ommen, G.-J.B., de Kimpe, S., Ferlini, A., et al. (2010). Preclinical PK and PD studies on 2′-O-methyl-phosphorothioate RNA antisense oligonucleotides in the *mdx* mouse model. Mol. Ther. *18*, 1210–1217.
8. Kurrikoff, K., Vunk, B., and Langel, Ü. (2021). Status update in the use of cell penetrating peptides for the delivery of macromolecular therapeutics. Expet Opin. Biol. Ther. *21*, 361–370.
9. Pei, D., and Buyanova, M. (2019). Overcoming endosomal entrapment in drug delivery. Bioconjugate Chem. *30*, 273–283.
10. Pei, D. (2022). How do biomolecules cross the cell membrane. Acc. Chem. Res. *55*, 309–318.
11. Qian, Z., Liu, T., Liu, Y.Y., Briesewitz, R., Barrios, A.M., Jhiang, S.M., and Pei, D. (2013). Efficient delivery of cyclic peptides into mammalian cells with short sequence motifs. ACS Chem. Biol. *8*, 423–431.
12. Qian, Z., LaRochelle, J.R., Jiang, B., Lian, W., Hard, R.L., Selner, N.G., Luechapanichkul, R., Barrios, A.M., and Pei, D. (2014). Early endosomal escape of a cyclic cell-penetrating peptide allows effective cytosolic cargo delivery. Biochemistry *53*, 4034–4046.
13. Qian, Z., Martyna, A., Hard, R.L., Wang, J., Appiah-Kubi, G., Coss, C., Phelps, M.A., Rossman, J.S., and Pei, D. (2016). Discovery and mechanism of highly efficient cyclic cell-penetrating peptides. Biochemistry *55*, 2601–2612.
14. Chen, K., and Pei, D. (2020). Engineering cell-permeable proteins through insertion of cell-penetrating motifs into surface loops. ACS Chem. Biol. *15*, 2568–2576.
15. Dougherty, P.G., Karpurapu, M., Koley, A., Lukowski, J.K., Qian, Z., Srinivas Nirujogi, T., Rusu, L., Chung, S., Hummon, A.B., Li, H.W., et al. (2020). A Peptidyl inhibitor that blocks calcineurin–NFAT interaction and prevents acute lung injury. J. Med. Chem. *63*, 12853–12872.
16. Sazani, P., Gemignani, F., Kang, S.-H., Maier, M.A., Manoharan, M., Persmark, M., Bortner, D., and Kole, R. (2002). Systemically delivered antisense oligomers upregulate gene expression in mouse tissues. Nat. Biotechnol. *20*, 1228–1233.
17. Zanta, M.A., Belguise-Valladier, P., and Behr, J.P. (1999 Jan 5). Gene delivery: a single nuclear localization signal peptide is sufficient to carry DNA to the cell nucleus. Proc. Natl. Acad. Sci. USA *96*, 91–96.
18. Gao, Q.Q., and McNally, E.M. (2015). The dystrophin complex: Structure, function and implications for therapy. Compr. Physiol. *5*, 1223–1239.
19. De Iaco, A., Planet, E., Coluccio, A., Verp, S., Duc, J., and Trono, D. (2017). DUX-family transcription factors regulate zygotic genome activation in placental mammals. Nat. Genet. *49*, 941–945.
20. Bao, B., Maruyama, R., and Yokota, T. (2016). Targeting mRNA for the treatmenet of facioscapulohumeral muscular dystrophy. Intractable Rare Dis. Res. *5*, 168–176.
21. Himeda, C.L., and Jones, P.L. (2022). FSHD therapeutic strategies: what will it take to get to clinic? J. Personalized Med. *12*, 865.
22. Tihaya, M.S., Mul, K., Balog, J., de Greef, J.C., Tapscott, S.J., Tawil, R., Statland, J.M., and van der Maarel, S.M. (2023). Facioscapulohumeral muscular dystrophy: the road to targeted therapies. Nat. Rev. Neurol. *19*, 91–108.
23. Hammond, S.M., Aartsma-Rus, A., Alves, S., Borgos, S.E., Buijsen, R.A.M., Collin, R.W.J., Covello, G., Denti, M.A., Desviat, L.R., Echevarría, L., et al. (2021). Delivery of oligonucleotide-based therapeutics: challenges and opportunities. EMBO Mol. Med. *13*, e13243.
24. Roberts, T.C., Langer, R., and Wood, M.J.A. (2020). Advances in oligonucleotide drug delivery. Nat. Rev. Drug Discov. *19*, 673–694.
25. Sugo, T., Terada, M., Oikawa, T., Miyata, K., Nishimura, S., Kenjo, E., Ogasawara-Shimizu, M., Makita, Y., Imaichi, S., Murata, S., et al. (2016). Development of antibody-siRNA conjugate targeted to cardiac and skeletal muscles. J. Contr. Release *237*, 1–13.
26. (2016). EXONDYS 51 (eteplirsen) [prescribing information]. (Sarepta Therapeutics, Inc). www.exondys51.com/modules/exondys/files/EXONDYS51PI.pdf
27. (2019). VYONDYS 53 (golodirsen) [prescribing information]. (Sarepta Therapeutics, Inc). https://www.vyondys53.com/assets/Vyondys53_Prescribing_Information.pdf
28. (2021). AMONDYS 45 (casimersen) [prescribing information]. (Sarepta Therapeutics, Inc). https://www.amondys45.com/Amondys45_(casimersen)_Prescribing_Information.pdf
29. (2020). VILTEPSO (Viltolarsen) [prescribing Information]. (NS Pharma, Inc). https://www.viltepso.com/prescribing-information
30. Sheikh, O., and Yokota, T. (2021). Restoring protein expression in neuromuscular conditions: A review assessing the current state of exon skipping/inclusion and gene therapies for Duchenne muscular dystrophy and spinal muscular atrophy. BioDrugs *35*, 389–399.
31. McDonald, C.M., Shieh, P.B., Abdel-Hamid, H.Z., Connolly, A.M., Ciafaloni, E., Wagner, K.R., Goemans, N., Mercuri, E., Khan, N., Koenig, E., et al. (2021). Open-label evaluation of eteplirsen in patients with Duchenne muscular dystrophy amenable to exon 51 skipping: PROMOVI trial. J. Neuromuscul. Dis. *8*, 989–1001.
32. Lim, K.R.Q., Nguyen, Q., and Yokota, T. (2020). DUX4 signalling in the pathogenesis of facioscapulohumeral muscular dystrophy. Int. J. Mol. Sci. *21*, 729.
33. Sazani, P., Kang, S.-H., Maier, M.A., Wei, C., Dillman, J., Summerton, J., Manoharan, M., and Kole, R. (2001). Nuclear antisense effects of neutral, anionic and cationic oligonucleotide analogs. Nucleic Acids Res. *29*, 3965–3974.
34. Sicinski, P., Geng, Y., Ryder-Cook, A.S., Barnard, E.A., Darlison, M.G., and Barnard, P.J. (1989). The molecular basis of muscular dystrophy in the *mdx* mouse: a point mutation. Science *244*, 1578–1580.

OMTN, Volume *33*

Supplemental information

The endosomal escape vehicle platform enhances delivery of oligonucleotides in preclinical models of neuromuscular disorders

Xiang Li, Mahboubeh Kheirabadi, Patrick G. Dougherty, Kimberli J. Kamer, Xiulong Shen, Nelsa L. Estrella, Suresh Peddigari, Anushree Pathak, Sara L. Blake, Emmanuelle Sizensky, Carmen del Genio, Arti B. Gaur, Mohanraj Dhanabal, Mahasweta Girgenrath, Natarajan Sethuraman, and Ziqing Qian

A

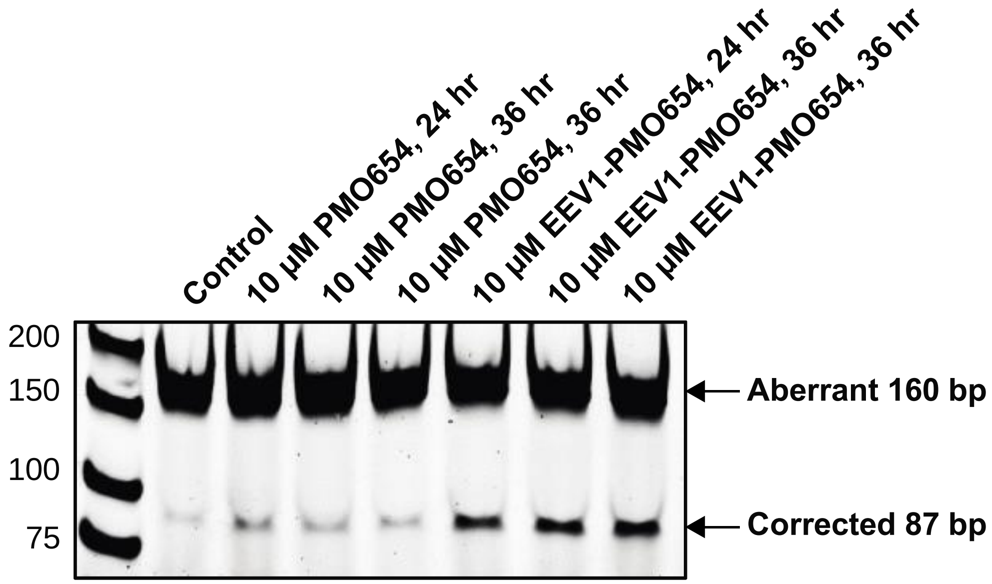

B

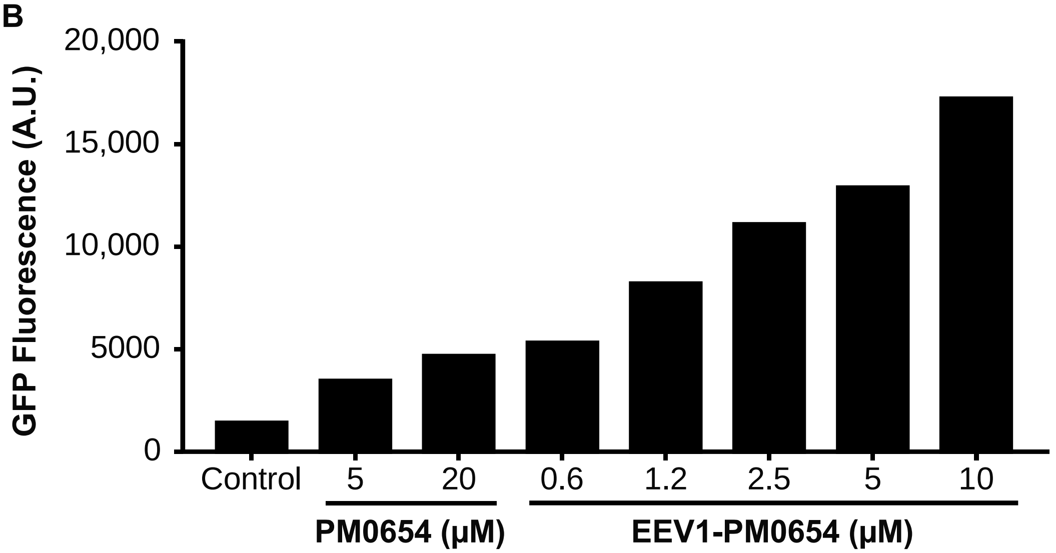

Figure S1. Evaluation of splice-switching activity for the EGFP pre-mRNA in HeLa EGFP-654 cells. (A) RT-PCR analysis of both corrected (87 bp) and uncorrected (160 bp) bands. (B) Restoration of EGFP fluorescence in HeLa EGFP-654 after 24-hour treatment and quantified by flow cytometry with 10,000 events per sample. Data were collected from a single experiment without replicates.

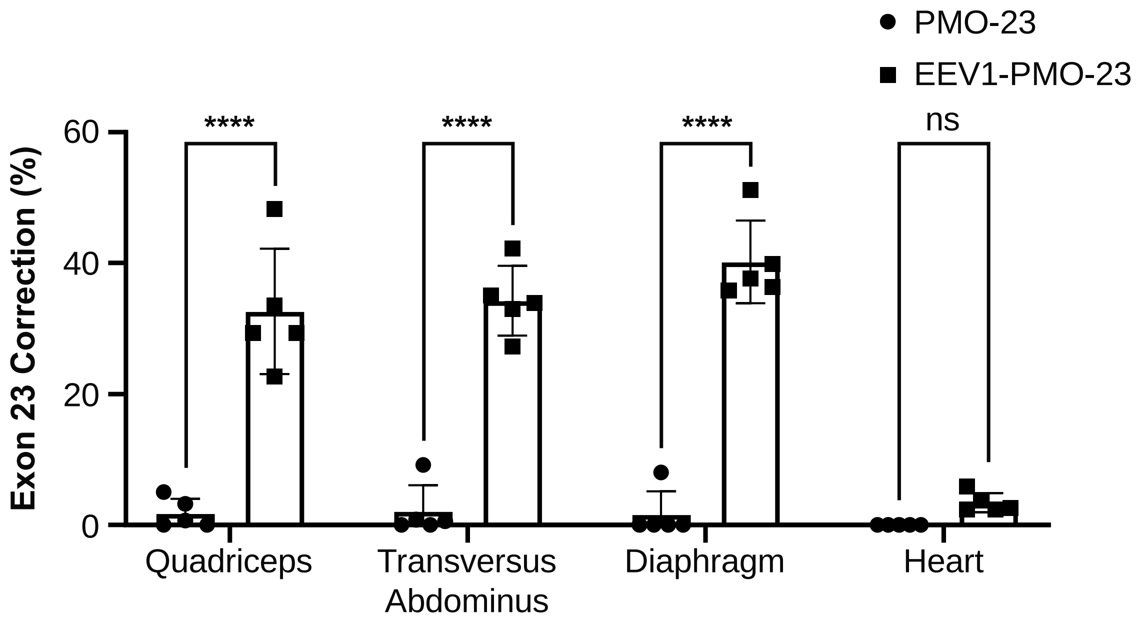

Figure S2. Exon 23–skipping induced by 30 mg/kg PMO or EEV1-PMO-23 (PMO equivalent) in C57BL/10 seven (7) days post-IV injection as quantified by RT-PCR

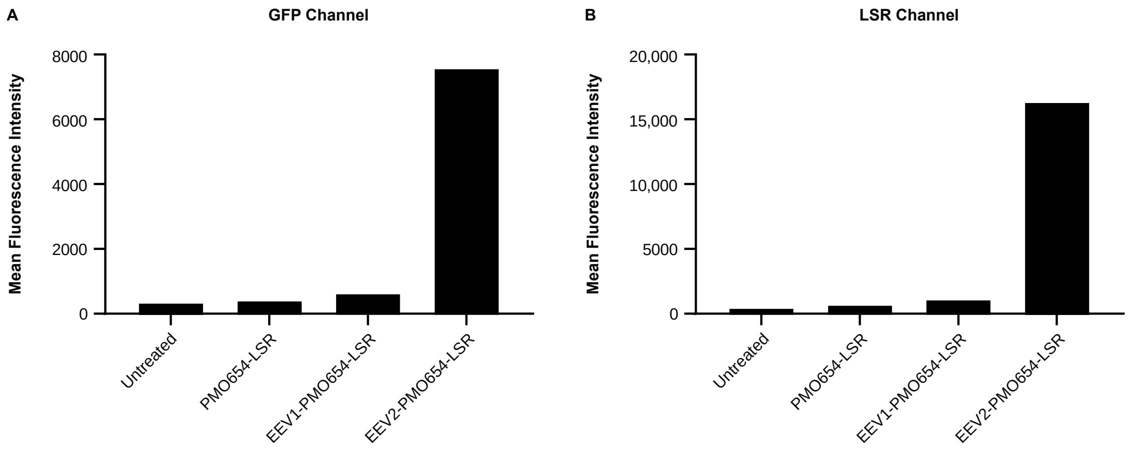

Figure S3. Flow cytometry analysis of both EGFP (A) and Lissamine Rhodamine (B) signals for HeLa-EGFP-654 cells treated with 2 µM Lissamine Rhodamine B (LSR) modified PMO654, EEV1-PMO654 and EEV2-PMO654 with 2000 events per sample. Data were collected from a single experiment without replicates
